# Determination of HER-2 status on FNAC material from breast carcinomas using *in situ* hybridization with dual chromogen visualization with silver enhancement (dual SISH)

**DOI:** 10.4103/1742-6413.70968

**Published:** 2010-10-11

**Authors:** Elsa Beraki, Torill Sauer

**Affiliations:** Department of Pathology, Oslo University Hospital, Ulleval, N-0424 Oslo, Norway

**Keywords:** Breast carcinoma, dual SISH, fine needle aspiration cytology, HER-2, *in situ* hybridization, liquid based material

## Abstract

During the last years, HER-2 status kits and protocols for chromogen visualization of hybridization signals have come on the market. The first generation using chromogen visualization used single color probes. The second generation, now emerging on the market, uses dual chromogen visualization. The aim of this study has been to test a new dual color chromogen kit (Ventana INFORM HER2 Dual Colour ISH Roche^®^) and compare the results with our in-house method(s). The material consisted primarily of cytological material from invasive breast carcinomas in 49 women. Dual SISH was done on all 49 cytological and histological specimens. The histological specimens were treated according to the manufacturer’s recommendations. The procedure was modified in several steps in order to adapt it to the cytological material. Hybridization failed in two cytological specimens. Dual SISH showed concordant results on cytological and histological material as to amplified/not amplified. The included cases had the same HER-2 expression in the invasive and the *in situ* components on histology. Four IDC showed HER-2 amplification (8.5%). Polysomy was found in two cases. All dual SISH results except for one concurred with the results of the in-house method(s) (1/47=2.1%). The dual SISH is suitable for cytological examination of HER-2 status. The protocol must be optimized for cytological material.

## INTRODUCTION

Breast carcinoma is a heterogeneous disease. The aggressiveness and prognosis of the tumors vary from low-grade carcinomas with an excellent prognosis to high-grade carcinomas with a poor prognosis. Prognostic and predictive markers such as estrogen and progesterone receptor status, HER-2 status and Ki-67 index are routinely evaluated in all carcinomas according to the recommendations of the Norwegian Breast Cancer Group (www.nbcg.no)[1] and national recommendations (www.helsedirektoratet.no/vp/multimedia/archive/00021/Nasjonalt_handlingsp_21559).[2] This is usually done on a preoperative biopsy or the surgical specimen. If necessary, all of the markers can also be evaluated on cytological material, but is most commonly used in metastatic settings where surgery or a biopsy is not feasible.[3] Cytological material is suitable for examining HER-2 using *in situ* hybridization (ISH)[3–6] and capping of tumor cell nuclei (meaning that in a histological section only part of, and not the whole nucleus, is represented) is not an issue. FISH (fluorescent *in situ* hybridization) protocols for HER-2 determination can normally be used on cytological material without or with only minor changes in the procedure for histological specimens.

During the last years, HER-2 status kits and protocols for chromogen visualization of hybridization signals have come on the market. These have been preferred in many pathology laboratories. The morphology is the same as in immunohistochemistry (IHC) and immunocytochemistry (ICC). The slides can be read using bright field microscopes and can be stored in the same way as the other cytological and histological specimens. The first generation using chromogen visualization used single color probes.[7–11] Kim *et al*,[5] demonstrated its usefulness in cytological specimens. The second generation, now emerging on the market, uses dual chromogen visualization.[12] The aim of this study has been to test a new dual color chromogen kit (Ventana INFORM HER2 Dual Colour ISH Roche^®^) and compare the results with our in-house method(s).

## MATERIALS AND METHODS

The material consisted primarily of cytological material from breast lesions in 50 women. All cases had been preoperatively investigated with FNAC. The morphological preoperative diagnoses were based on direct, air - dried and Giemsa stained smears. Additional liquid based material (primarily suspended in ThinPrep Cytolyt and stored in ThinPrep Preservcyt (Hologic™, Crawley, West Sussex, UK) was available for estrogen and progesterone receptor status as well as HER-2 status. One lesion was cytologically benign and was included as benign “control”. Histologically, it was a benign phyllodes tumor. *In situ* hybridization failed in two cytological specimens. This left us with concordant cytological and histological material from 47 invasive carcinomas.

The surgical specimens had been routinely processed and diagnosed, including HER-2 status. In addition to IHC (Ventana INFORM^®^), FISH (previous in-house method, Ventana INFORM FISH^®^) and (single probe) SISH (current in-house method, Ventana INFORM SISH^®^) were used. IHC and FISH were in-house methods in the department from 2003 to 2008. Single probe SISH then replaced FISH as in-house method. The material for this study was collected during 2008 - early 2009. Until late 2008, both IHC and ISH were done routinely on all new primary breast carcinomas for QC purposes. These comprised 29 cases (14 cases IHC + single probe SISH; 19 cases IHC + single probe FISH). A shorter period using single probe SISH as the primary test followed (14 cases) before the department started to use IHC as the primary test and with additional SISH according to national guidelines (that is when IHC is 2+).

Dual SISH (Ventana INFORM HER2 Dual Colour ISH, Roche^®^) was done on all 47 cytological and histological specimens. The histological specimens were treated according to the manufacturer’s recommendations. The procedure was modified in several steps in order to adapt it to the cytological material [Table 1]. This was done prior to the study investigation. Strong and clearly identifiable signals for both HER-2 gene and CEP17 were demanded for optimization.

**Table 1:** Procedure for dual SISH and red ISH on cytological material on the BenchMark XT IHC/ISH staining machine

Day 1
Fix slides in 4% neutral buffered formalin (NBF) for 2 h at the bench
Wash slides in Tris buffered saline (TBS) 2×5 min
Load slides in the staining machine and start the protocol for cytological material
Cell conditioning: Incubate slides in mild followed by standard cell conditioning buffer (CC2) for 8 min each.
After enzyme (protease 3) incubation for 8 min, warm up slides to 47°C prior to hybridization
Incubate slides with HER-2 DNA probe and hybridize for 6 h at 47°C
Warm up slides up to 67°C and incubate in SISH stringent wash buffer 3×8 min.
Incubate slides with Red ISH-probe (Chr17 Probe) at 44oC for 4 min and further for 2 h.
Wash slides in Red ISH stringent wash at 59°C for 3×8 min.
Counterstain slides with hematoxylin II for 8 min followed by post counterstain with Bluing reagent for 4 min.
Day 2
Wash slides in warm tap water for 10 min.
Air dry slides completely (e.g. 15 min at 56°C)
Coverslip with non-alcoholic/non-xylen based mounting medium

HER-2 scoring was identical for cytological and histological specimens and was according to the manufacturer’s instructions. A ratio HER-2/CEP17 of > 2.2 was regarded as amplification. A ratio of less than 1.8 was considered not amplified and 1.8 – 2.2 as equivocal. Signals in at least 20 non-overlapping nuclei in two different fields were counted. Alternatively, in overlapping groups, the total number of nuclei and signals were counted and a mean calculated. A mean signal number and a ratio between the HER-2 and CEP17 were calculated. Polysomy was defined as a mean signal number of > 3 and a ratio of < 1.8.

Tumor subtype, grading as well as ER/PgR and routinely performed HER-2 status were retrieved from the pathology files. At the time of the study, cut-off for a positive ER/PgR was 10%. This has since been changed to 1%.

## RESULTS

There were 39 ductal (IDC), 5 lobular (ILC), 2 mucinous and 1 invasive papillary carcinoma. Of these, 11 were grade 1, 27 were grade 2 and 9 were grade 3. Estrogen receptor (ER) was strongly positive (that is in > 50% of the tumor cells) in 40 cases and negative in 7 cases. Progesterone receptor (PgR) was negative in 15 cases, positive in < 50% of the tumor cells in 12 cases and positive in > 50% of the tumor cells in 20 cases. All ILC and mucinous carcinomas as well as the papillary carcinoma were ER/PgR positive. Tumor sizes were as follows: pT1b =8, pT1c =16, pT2 =16 and pT3 =7. Axillary lymph node metastases were found in 25 cases, whereas 22 cases were N0.

The red (CEP17) and silver (HER-2 gene) chromogen signals could be read at ×40 magnification, but were easier to evaluate under oil immersion (×100) [Figure 1a and b]. The optimal signal size and “sharpness” depended on the post hybridization washing conditions and were different for the two chromogens. The final procedure represented a compromise allowing both signals to be read easily [Table 1]. The differences in the cytological and histological procedures are highlighted in Table 2. As can be seen, a number of steps were modified, including type of buffer, hybridization temperature and stringent wash temperature. CC2 buffer gave better results as RB and the pretreatment could be somewhat shorter than for histological specimens. Likewise, the optimal hybridization and stringent wash temperatures were somewhat lower when using cytological preparations.

**Figure 1a:**
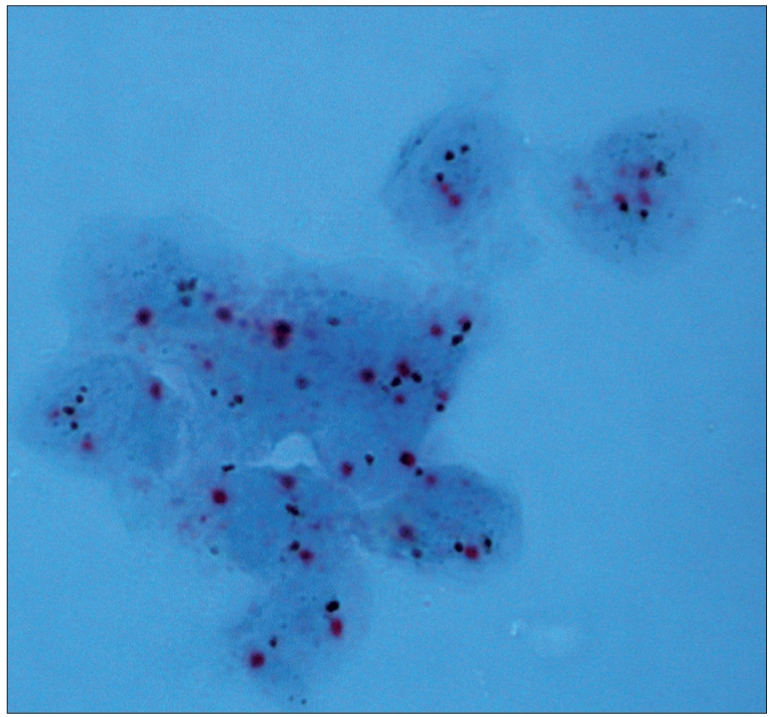
Dual color SISH. Magnification × 1000. Cytological specimen, direct smear. Non-amplified with two signals of CEP17 (red) and HER-2 gene (black) per nucleus

**Figure 1b:**
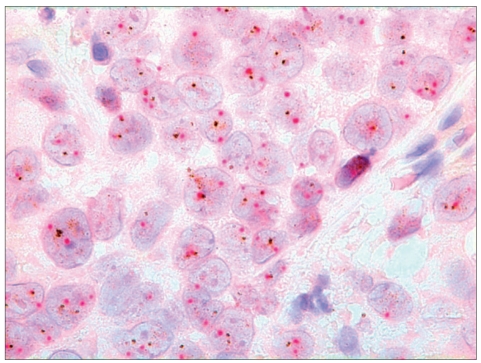
Dual color SISH. Magnification × 1000. Histological specimen. Non-amplified with two signals of CEP17 (red) and HER-2 gene (black) per nucleus

**Table 2:** Comparison of cytological and histological protocols for Auto Dual SISH staining

*Cytological protocol*	*Histological protocol*
Cell conditioning:- Cell conditioning buffer (CC2)	Cell conditioning:- Reaction buffer (RB)
Citrate-buffer with pH 6.0 and containing	Tris-buffer with pH 7.6 -7.8 and containing
Ethylene glycol	Tris (hydroxy methyl) aminomethane
Dodecyl sodium sulfate	Acetic acid
Hydrous citric acid	Proclin
Sodium metabisulfate	
Mild CC2 8 min	Mild RB 8 min
Standard CC2 8 min	Standard RB 12 min
	Extended RB 8 min
SISH hybridization temperature 47°C	SISH hybridization temperature 52°C
SISH stringent wash temperature 67°C	SISH stringent wash temperature 72° C

Dual SISH showed 96% concordant results on cytological and histological study material. An equivocal ratio was found in two cytological specimens (1.94 and 1.97). These two cytological cases were listed as non-amplified. The corresponding histological ratios were 1.51 and 1.52, respectively. No case was found to be amplified on the cytological specimen and non-amplified on histological specimen or vice versa. The included cases had the same HER-2 expression in the invasive and the *in situ* components on histology. Four IDC showed HER-2 amplification (8.5%) on dual SISH [Figure 2a and b]. Corresponding in – house HER-2 status was based on IHC + FISH in one case, on IHC + SISH in one case, and on IHC 3+ alone in two cases. Polysomy [Figure 3] was found in 2 cases. All dual SISH study results (both cytological and histological) except for one concurred with the routine HER-2 status results of the in-house method(s) (1/47=2.1%). The discordant case was amplified on both cytological and histological specimens using dual SISH with ratios of 4.18 and 3.64, respectively. Routine IHC had been 2+, but the current in-house SISH showed only 1-2 signals per nucleus of both HER-2 gene and CEP17. An overview of signal counts and ratios is shown in Table 3.

**Figure 2a:**
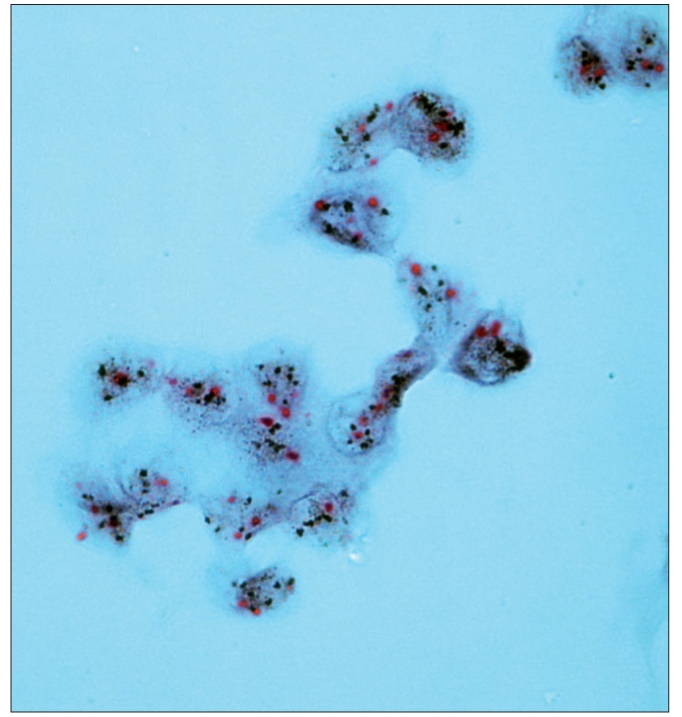
Dual color SISH. Magnification × 1000. Cytological specimen, liquid based preparation. Two CEP17 (red) signals and from 5 to > 10 HER-2 gene signals (black) per nucleus. Amplification.

**Figure 2b:**
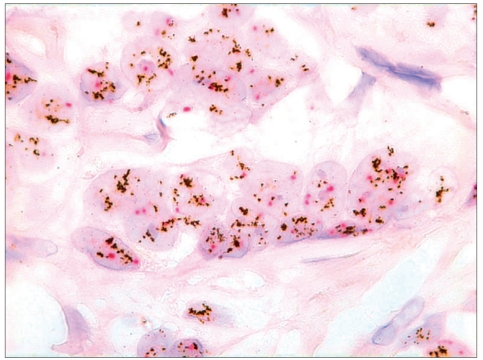
Dual color SISH. Magnification × 1000. Histological specimen. Two CEP17 (red) signals and a highly increased number of HER-2 gene signals (black) per nucleus. Amplification.

**Figure 3:**
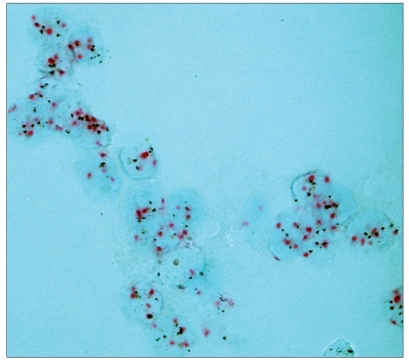
Dual color SISH. Magnification × 1000. Cytological specimen. Liquid based preparation. Polysomy with increased number of both CEP17 (red) and HER-2 gene (black) per nucleus

**Table 3:** Overview of signal counts and ratios

	*Mean*	*Median*	*Minimum*	*Maximum*
Number of HER-2 gene signals CYT/HIST non-amplified (=43)	2.09/1.76	1.95/1.6	1.26/1.08	4.1/3.25
Number of CEP17 signals CYT/HIST non-amplified	1.84/1.54	1.75/1.45	1.13/1	3.05/2.95
Ratio HER-2/CEP17 CYT/HIST non-amplified	1.14/1.15	1.11/1.14	0.74/0.68	1.97/1.54
Number of HER-2 gene signals CYT/HIST amplified (=4)	10.25/8.69	10.5/7.83	8/5.8	12/13.29
Number of CEP17 signals CYT/HIST amplified	2.21/1.98	2.27/2	2/1.8	2.3/2.1
Ratio of HER-2/CEP17 CYT /HIST amplified	4.61/4.37	4.61/3.82	4/3.2	5.22/6.65

## DISCUSSION

The ASCO/CAP guidelines for HER-2 testing[13] state that “If laboratories choose to use alternative fixatives other than buffered formalin, the laboratory is obligated to validate that fixative’s performance against the results of testing of the same samples fixed also in buffered formalin and tested with the identical HER2 assay, and concordance in this situation must also be 95%.” A FAQ web site[14] to these guidelines answer the question “Do the guidelines exclude HER2 testing of cytology specimens (fluids and aspirates) that have been fixed in 95% ethanol rather than formalin?” as follows: “Since cytology specimens are not ordinarily fixed in formalin, that sentence is not directly applicable, but labs performing HER2 testing on such specimens must document that they validated their methods and achieved acceptable concordance, in this case by comparing staining of alcohol fixed cytology specimens with routinely processed (formalin-fixed) tissue sections”. The complete concordance between dual color SISH results on cytological and histological specimens in this study as to amplification/no amplification as well as a concordance of 97.9% with the in-house method(s) indicates that this method and protocol is well suited to investigate HER-2 status on cytological material.

In contrast to FISH, where the histological protocol can be used practically unchanged for cytological specimens, the dual SISH method required adjustment of several steps in the protocol. The first crucial step is fixation. The primary fixation of cytological specimens is usually alcohol-based (ethanol or methanol) or acetone/acetic acid. Cross-linking is thus not occurring and demasking as when using formalin fixation is not necessary. However, formalin may also be used for fixation of cytological material. Several articles have reported different fixation methods for optimal sensitivity of ICC[15–19] and some have concluded that formalin is a good choice of fixative for ICC.[17] The same applies to ISH on cytological material. Here also, formalin fixation is a good option.[20]

Primary or post fixation of cytological material requires demasking, but usually much less than histological material. In this study, the specimens were post fixed in 10% formalin for 2h. Shorter or longer fixation periods resulted in weak or no gene and/or CEP17 signals.

Type of buffer is known to influence the results in immunocyto- and histochemistry and give anything from a false negative or weak positive reaction to a strong positive staining. In this study, optimal type of buffer and time for preconditioning of cytological material was different compared to the standard histological procedure given by the manufacturer. CC2 is a citrate buffer with PH 6 whereas RB is a Tris buffer with a higher of PH 7.6-7.8. As these modifications are not necessary using a FISH procedure, the differences may relate mainly to the reaction of the chromogen substances and not to the hybridization step.

Mean signal counts were higher in the cytological specimens as expected, because there is no capping of the nuclei. The ratios, however, were virtually the same in the concurrent cytological and the histological specimens with the exception of two cases with an equivocal ratio on cytological specimen and a clearly non-amplified ratio on the histological specimen. The number of amplified and polysomic cases was somewhat low. This is probably due to inclusion bias.

Chromogen ISH has a number of advantages. The morphology is familiar to what we see and evaluate in our everyday diagnostics, both in cytological and histological specimens. The cytomorphology is recognizable and the risk of including benign cells in our evaluation is minimal. The signals can be read in bright field microscope and the specimens may be stored alongside with the diagnostics slides in the department.

One of the objections to evaluating the results of ancillary studies on cytological material from primary breast carcinomas is that there may be a mixture of cells from the invasive component and from a component of ductal carcinoma *in situ*. This does not preclude testing for HER-2 (or ER/PgR) in metastatic settings, provided the institutions have adequate methods. In this study, the included cases had the same HER-2 expression in the invasive and the *in situ* components on histology.

In conclusion, cytological material is suitable for HER-2 examination using dual chromogen visualization with silver enhancement (Dual SISH) *in situ* hybridization (Ventana INFORM HER2 Dual Colour ISH Roche^®^). The protocol must be modified and optimized for cytological material.

## COMPETING INTEREST STATEMENT BY ALL AUTHORS

No competing interest to declare by any of the authors.

## AUTHORSHIP STATEMENT BY ALL AUTHORS

Each author acknowledges that this final version was read and approved. All authors qualify for authorship as defined by ICMJE http://www.icmje.org/#author Each author participated sufficiently in the work and takes public responsibility for appropriate portions of the content of this article.

## ETHICS STATEMENT BY ALL AUTHORS

This study was conducted with approval from institutional Review Board (IRB) (or its equivalent) of all the institutions associated with this study.

## EDITORIAL / PEER-REVIEW STATEMENT

To ensure integrity and highest quality of CytoJournal publications, the review process of this manuscript was conducted under a double blind model(authors are blinded for reviewers and reviewers are blinded for authors)through automatic online system.
